# A loss‐of‐function mutation p.T256M in NDRG4 is implicated in the pathogenesis of pulmonary atresia with ventricular septal defect (PA/VSD) and tetralogy of Fallot (TOF)

**DOI:** 10.1002/2211-5463.13044

**Published:** 2021-01-09

**Authors:** Jiayu Peng, Qingjie Wang, Zhuo Meng, Jian Wang, Yue Zhou, Shuang Zhou, Wenting Song, Sun Chen, Alex F. Chen, Kun Sun

**Affiliations:** ^1^ Department of Pediatric Cardiology Xinhua Hospital School of Medicine Shanghai Jiao Tong University Shanghai China; ^2^ Department of Pediatric Cardiology The Second Affiliated Hospital and Yuying Children's Hospital of Wenzhou Medical University Zhejiang China; ^3^ Institute of Cardiovascular Development and Regeneration Xinhua Hospital School of Medicine Shanghai Jiao Tong University Shanghai China; ^4^ Department of Cardiology Xinhua Hospital School of Medicine Shanghai Jiao Tong University Shanghai China

**Keywords:** cardiac myocytes, NDRG4, p27, PA/VSD, proliferation, TOF

## Abstract

Pulmonary atresia with ventricular septal defect (PA/VSD) is a rare congenital heart disease (CHD) characterized by a lack of luminal continuity and blood flow from either the right ventricle or the pulmonary artery, together with VSDs. The prevalence of PA/VSD is about 0.2% of live births and approximately 2% of CHDs. PA/VSD is similar to tetralogy of Fallot (TOF) in terms of structural and pathological characteristics. The pathogenesis of these two CHDs remains incompletely understood. It was previously reported that N‐myc downstream‐regulated gene (NDRG)4 is required for myocyte proliferation during early cardiac development. In the present study, we enrolled 80 unrelated patients with PA/VSD or TOF and identified a probably damaging variant p.T256M of NDRG4. The p.T256M variant impaired the proliferation ability of human cardiac myocytes (hCM). Furthermore, the p.T256M variant resulted in G1 and G2 arrest of hCM, followed by an increase in p27 and caspase‐9 expression. Our results provide evidence that the p.T256M variant in NDRG4 is a pathogenic variant associated with impaired hCM proliferation and cell‐cycle arrest and likely contributes towards the pathogenesis of PA/VSD and TOF.

## Graphical abstract

N‐myc downstream‐regulated gene (NDRG)4 is widely expressed in human embryonic hearts. The p.T256M variant in NDRG4 resulted in G1 and G2 arrest of human cardiac myocytes, thereby impairing their proliferative ability, which likely contributes towards the pathogenesis of pulmonary atresia with ventricular septal defect and tetralogy of Fallot.

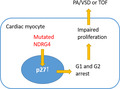

Pulmonary atresia with ventricular septal defect (PA/VSD) is a rare complex type of congenital heart disease (CHD) characterized by a lack of luminal continuity and blood flow from either the right ventricle and the pulmonary artery, together with VSD [1, 2, 3]. The prevalence of PA/VSD is about 0.2% of live births and approximately 2% of CHDs. In addition, PA/VSD is also considered to be one of the most complex and unmanageable CHD [3, 4]. PA/VSD is similar to tetralogy of Fallot (TOF) in structural and pathological characteristics. PA/VSD and TOF are both characterized by a lack of continuity of right ventricle outflow tract and pulmonary blood supply, together with VSD. The difference is the severity of obstruction of right ventricle outflow tract. Therefore, it is moderately accepted that PA/VSD is the most severe type of TOF [3, 5, 6].

However, the pathogenesis of these two CHDs still remains to be investigated. Previous evidence has indicated that cardiomyocyte proliferation shapes cardiac structures [7]. The first cardiomyocytes to emerge during development form primitive hollowed chambers [8]. As embryos mature to juvenile and adult stages, the regulated division of cardiomyocytes is responsible for the formation, growth and sculpting of mature cardiac structures, such as the fingerlike ventricular trabeculae and atrial pectinate, as well as the layered compact muscle of chamber walls [8]. Therefore, the impaired cardiomyocyte proliferation will result in heart defects [9, 10, 11], including outflow tract anomalies. [12] Several molecules and pathways are involved in regulating cardiac myocyte proliferation: cell‐cycle regulators, noncoding RNA, developmental gene programs and cell–cell interactions [13]. The cell cycle of cardiomyocyte is accompanied by downregulation of numerous essential cell‐cycle factors and upregulation of cell‐cycle inhibitors [8]. In addition, some mutations in genes involved in the regulation of cardiomyocyte proliferation can also affect the cardiomyocyte proliferation. For example, following a mutation in REPTIN [also called RUVBL2 (RuvB‐like AAA ATPase 2)], a member of the AAA + ATPase family and a component of the INO80 ATPase complex, its ATPase activity leads to cardiac muscle hyperplasia in zebrafish [14].

The N‐myc downstream‐regulated gene (NDRG) family of proteins consists of four members, NDRG1–4, which are well conserved through evolution. Expression of all NDRG family members appears to correlate with progressive stages of differentiation, increasing from birth to adulthood. NDRG4, also known as BDM1 and SMAP‐8, is one of four members of the NDRG family and is involved in cell proliferation, differentiation, development and stress [15]. Previous studies have shown that NDRG4 is highly expressed in the heart [16], moderately expressed in vascular smooth muscle cells [17] and involved in cardiac development [18]. A previous study reported that NDRG4 is required for normal myocyte proliferation during early cardiac development [19]. Furthermore, another study suggested that Bves and NDRG4 regulated directional epicardial cell migration, which is of great importance for cardiac morphogenesis [20].

The present study aimed to further investigate the genetic etiology of PA/VSD and TOF. Accordingly, we used whole‐exome sequencing and identified a candidate damaging mutation in NDRG4 from 80 unrelated patients with PA/VSD or TOF and a population‐matched control cohort of 100 healthy children. Further function experiments showed that the p.T256M variant in NDRG4 may be involved in the pathogenesis of PA/VSD and TOF by impairing regulation of cardiomyocyte proliferation.

## Materials and methods

### Study population

We recruited unrelated patients with PA/VSD (*n* = 60) or TOF (*n* = 20) and 100 healthy children without heart diseases as controls from Shanghai Xinhua Hospital (Shanghai, China) and Shanghai Children Medical Center (Shanghai, China). All of the patients were diagnosed by cardiac catheterization, echocardiogram or surgery. The study was approved by the Ethics Committee of Xinhua Hospital and the study design conformed to the guidelines set by the Declaration of Helsinki.

### Ethical statement

All assessments were performed under the approval of the Medical Ethics Committee of Xinhua Hospital and Shanghai Children Medical Center (XHEC‐C‐2019‐083). All experiments were performed in accordance with the approved guidelines. We obtained written informed consent from all participants (or their parents if the children were too young to consent by themselves).

### Cells and samples

The genomic DNA for gene sequencing was extracted from the blood sample from patients or healthy controls. The human cardiomyocytes (hCM) cell line was purchased from Beina Biology (Beijing, China) (BNCC337719). Human embryo heart samples were obtained from pregnancy termination.

### Gene sequencing and variant analysis

Gene sequencing and variant analysis were performed as described previously [21, 22]. Briefly, we used the QIAamp DNA Blood Mini Kit (Qiagen, Hilden, Germany) to extract the genomic DNA of participants in accordance with the manufacturer's instructions. Then, the extracted DNA was stored at −80 °C. Target sequencing was conducted by the Illumina HiSeq 4000 platform (Illumina Inc., San Diego, CA, USA) for variants in NDRG4 (GenBank accession number NC_000016.10, NM_020465.4). The candidate variant was confirmed by Sanger sequencing. Then we designed primers for PCR amplification of NDRG4. We used several criteria including sift (http://sift.jcvi.org/www/SIFT_enst_submit.html) and polyphen‐2 (http://genetics.bwh.harvard.edu/pph2/) to predict the effects of nonsynonymous variants. When the score was ≥ 0.85 in polyphen‐2 or ≤ 0.05 in sift, the amino acid substitutions were predicted as damaging. In the present study, variants with a minor allele frequency [23] < 0.5% were defined as rare [24].

### Gene expression analysis of the microarray datasets

Human embryo heart samples at Carnegie stage (CS)11–15 were obtained from pregnancy termination at the Shanghai Xinhua Hospital. Then, we extracted the total RNA using the TissueLyser II (Qiagen) and an RNeasy MinElute Cleanup Kit (Qiagen). A transcriptome array was performed to detect gene expression levels at different developmental stages. Raw data were normalized using affymetrix u133 plus 2.0 Array software (Thermo Fisher Scientific, Waltham, MA, USA) and the signal value calculated by log_2_ transformation was the normalized signal value [22].

### Multiple NDRG4 protein sequence alignment

NDRG4 protein sequences from various species including *Homo sapiens* (human), *Mus musculus* (house mouse), *Xenopus tropicalis* (frog), *Rattus norvegicus* (rat), *Danio rerio* (zebra fish), *Oryctolagus cuniculus* (rabbit) and *Bos taurus* (cattle) were downloaded the from Universal Protein (UniProt) database (http://www.uniprot.org). Sequences alignment was performed using clustalw software (http://www.clustal.org).

### Plasmid construction and site‐directed mutagenesis

The NDRG4 cDNA plasmid, which was cloned into pCMV6‐entry vectors with a MYC tag, was purchased from OriGene (Rockville, MD, USA). Mutated primers were designed to amplify human NDRG4 cDNA according to the protocol provided with the QuikChange Site‐Directed Mutagenesis Kit (Stratagene, La Jolla, CA, USA). The mutated site was confirmed by Sanger sequencing [21, 22].

### Cell cultures and transfection

Human cardiac myocytes were maintained in Dulbecco’s modified Eagle's medium (HyClone, Logan, UT, USA) with 10% FBS (MP Biomedicals, Carlsbad, CA, USA) and 1% penicillin‐streptomycin (Gibco, Thermo Fisher Scientific). pCMV6‐NDRG4 including wild‐type (WT) and variant were transfected into hCM with FuGENE HD (Promega, Madison, WI, USA) in accordance with the manufacturer’s instructions.

### Quantitative RT‐PCR (qRT‐PCR)

Human cardiac myocytes were seeded in 12‐well plates then plasmids were transfected into hCM. Twenty‐four hours after transfection, we harvested the cells. Total RNA was extracted with EZ‐press RNA Purification Kit (EZBioscience, Roseville, MN, USA). Then, we used cDNA Synthesis Kit (Yeasen, Shanghai, China) to perform the reverse transcription of cDNA. After that, we conducted qRT‐PCR using qPCR SYBR Green Master Mix (Yeasen) on an Applied Biosystems 7500 system (Applied Biosystems, Foster City, CA, USA). The relative quantification of mRNA expression was determined using the 2^−▵▵Ct^ method [25] and glyceraldehyde‐3‐phosphate dehydrogenase (GAPDH, human) was used as an internal control [21, 22]. The primer sequences of NDRG4, GAPDH, P27, cyclin D1, cyclin E, caspase 3 and caspase‐9 are listed in Table S1.

### Western blotting

Human cardiac myocytes were transfected with 1 μg of WT and variant plasmid DNA. Then, we harvested the cells 48 h after transfection. We used western and immunoprecipitation lysis buffer (Beyotime, Shanghai China) with protease and phosphatase inhibitor cocktail (Beyotime) to lysis cells. The proteins were subjected to 10% SDS/PAGE and were then transferred onto nitrocellulose membranes (Millipore, Burlington, MA, USA) and immunostained with rabbit anti‐NRDG4 antibody (dilution 1 : 500; Novus Biologicals, Centennial, CO, USA), rabbit anti‐GAPDH antibody (dilution 1 : 5000; Proteintech, Chicago, IL, USA) rabbit anti‐P27 antibody (dilution 1 : 1000; Cell Signaling Technology, Beverly, MA, USA) at 4 °C overnight. The membranes were incubated with HRP‐conjugated AffiniPure Goat Anti‐Rabbit IgG(H + L) secondary antibody (dilution 1 : 10 000; Proteintech) [21, 22]. All the raw immunoblots are available in the supplement (Figure S1).

### Tissue collection and immunohistochemistry

Human embryos of CS13 were acquired after medical termination of pregnancy at Shanghai Xinhua Hospital. Embryos were fixed for 24 h in 4% paraformaldehyde and then were sent to Servicebio (Wuhan, China) to embed and section. Next, sections were stained with a primary rabbit anti‐NDRG4 antibody (dilution 1 : 500; Novus), followed by a goat anti‐rabbit secondary antibody, and then the signals were visualized using diaminobenzidine (Servicebio) [21, 22].

### Cell proliferation analysis

Cell Counting Kit‐8 (CCK‐8; Beyotime) was used to analyze the proliferation of hCM cells. Briefly, cells were transfected with WT, variant NDRG4 plasmid or their negative control vectors for 24 h. After digestion with 0.25% trypsin (Gibco), the cells were collected and seeded into 96‐well plates (3 × 10^3^ per well), followed by incubation in an incubator at 37 °C with 5% CO_2_. Then, cells were maintained at 37°C for 2 h after the addition of CCK‐8 solution at 0, 24, 48 and 72 h. The optical density of each well was tested at 450 nm with a microplate reader (BioTek Inc., Winooski, VT, USA).

### Flow cytometry

To evaluate cell cycle, flow cytometry was performed 48 h after transfection. hCM cells were seeded in six‐well plates. Cells were fixed in 70% cold ethanol and stained with propidium iodide solution (Beyotime). All labeled cells were analyzed on a flow cytometry instrument (Beckman Coulter, Fullerton, CA, USA).

### Immunofluorescence assay

Human cardiac myocytes cells were seeded onto a 12‐well plate covered with slips and then transfected with WT or variant plasmid DNA. Cells were harvested 24 h after transfection. Cells were incubated with rabbit anti‐P27 antibody (dilution 1 : 800; Cell Signaling Technology) diluted in NaCl/P_i_ containing 5% goat serum (Beyotime) and 0.2% Triton X‐100 at 4 °C overnight, followed by incubation with Cy3‐conjugated goat anti‐rabbit secondary antibody (dilution 1 : 300; Jackson ImmunoResearch, West Grove, PA, USA). Cell nuclei were stained using 4',6‐diamidino‐2‐phenylindole (Servicebio). Image analysis [21, 22] was condicted using a SP8 microscope (Leica Microsystems, Wetzlar, Germany).

### Statistical analysis

Each assay was performed for four to five independent biological replicates. The data are reported as the mean ± SEM. Statistical differences were evaluated by one‐way analysis of variance or a two‐tailed unpaired *t*‐test. *P* < 0.05 was considered statistically significant.

## Results

### Identification of a NDRG4 mutation (c.767C>T) in PA/VSD and TOF patients

**Fig. 1:**
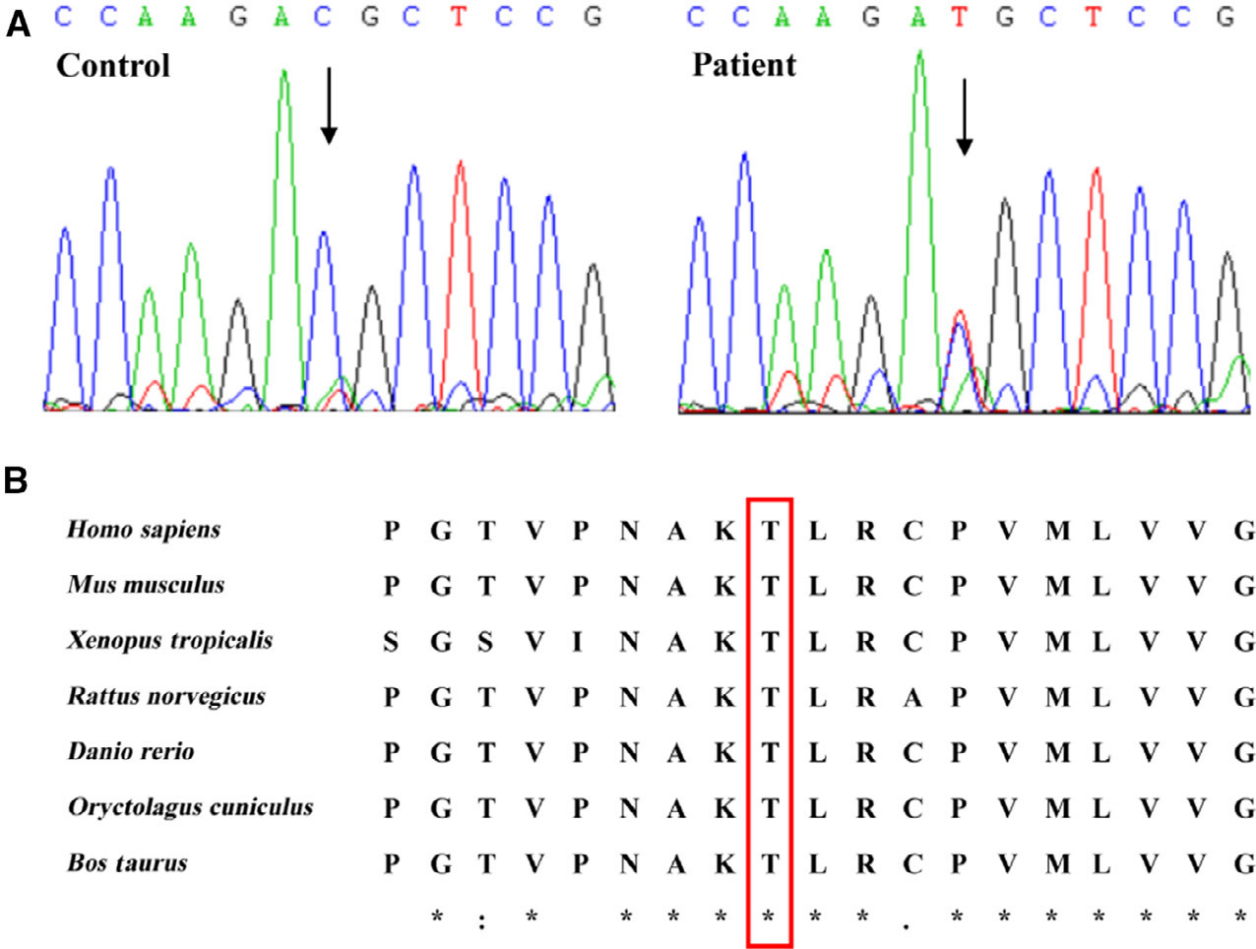
Characteristics of the mutation c.767C>T in NDRG4. (A) Sequence chromatograms of NDRG4 variants in patients. Arrows indicate heterozygous nucleotide changes. (B) Homology analysis of the 256T position of NDRG4 protein across different species.

A heterozygous missense variant in NDRG4 was identified in three unrelated PA/VSD and TOF patients out of the 80 unrelated patients with PA/VSD or TOF, with the variant being absent from the 100 controls in our cohort (Fig. 1A). The NDRG4 mutation, p.T256M (NM_020465: c.767C>T, rs144494221) was found in the ExAC database (http://exac.broadinstitute.org) and the allelic frequency was 0.001 (Table 1).

**Table 1:** Clinical information and variant characteristics of NDRG4 in patients.

Patient	Gender	Age	Cardiac phenotype	Nucleotide change	Amino acid change	dbSNP ID	SIFT	PolyPhen‐2	ExAC allele frequency
1	Female	6 months	PA, VSD, patent foramen ovale, patent foramen ovale	c.767C>T	T256M	rs144494221	0.01	0.934	0.001
2	Female	7 months	TOF, patent foramen ovale						
3	Female	1 year	TOF, patent foramen ovale, left superior vena cava						

The variation site in the present study was highly conserved in vertebrates, as shown in the multiple NDRG4 protein alignments (Fig. 1B), indicating that this variant was very important and might result in NDRG4 gene function alterations.

### Expression of NDRG4 protein in the human embryo

**Fig. 2:**
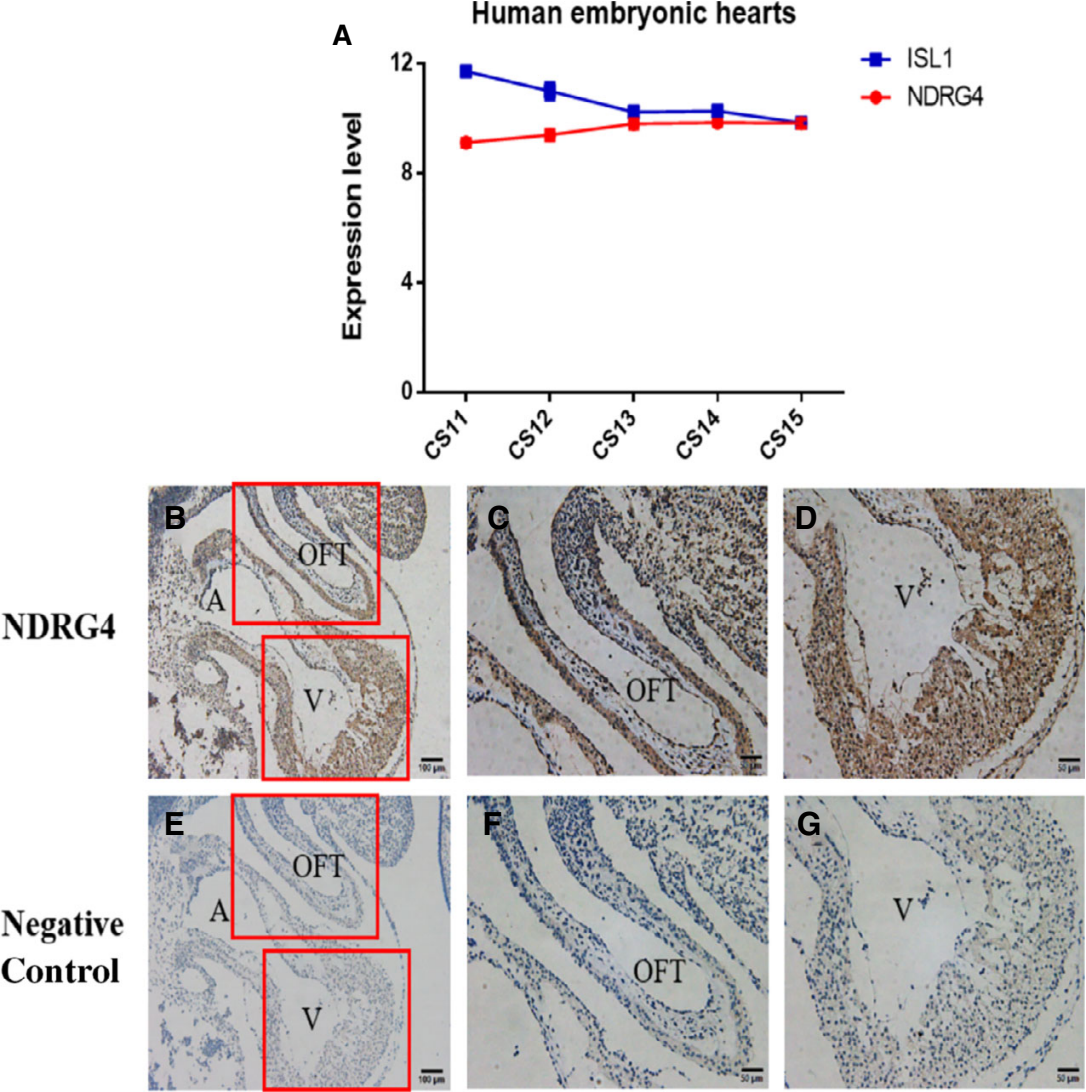
mRNA and protein expression levels of NDRG4 in human embryos. (A) The mRNA expression levels of NDRG4 and ISL1 in the same samples were investigated in human embryonic hearts (*n* = 3). (B–G) Immunohistochemistry of NDRG4 in human embryos at CS13. (B–D) Negative control. (E–G) WT NDRG4. ISL1, Islet‐1; OFT, outflow tract; A, atrium; V, ventricle. Scale bar in (B, E) = 50 μm, Scale bar in (C, D, F, G) = 20 μm.

NDRG4 has been reported as being localized in both epicardial cells and cardiac myocytes in E14.5 murine cardiac tissue [20]. However, NDRG4 expression has not been identified in the human embryo. Therefore, we collected human embryonic hearts from CS11 to CS15 and performed gene expression analysis using affymetrix u133 plus 2.0 Array software. The mRNA expression levels of NDRG4 were represented by the mean ± SEM of the sample expression levels. Our analysis revealed that NDRG4 was highly expressed throughout these development stages (Fig. 2A), approximately as high as Islet‐1 (Isl1), which makes a substantial contribution to the embryonic heart [18]. Then, we carried out immunohistochemistry in human embryos at CS13 and our results showed that NDRG4 was widely expressed in the atrium, ventricle and outflow tract [26] (Fig. 2B–G). These data indicated that NDRG4 might be involved in human cardiac development.

### Detection of NDRG4 variant expression

**Fig. 3:**
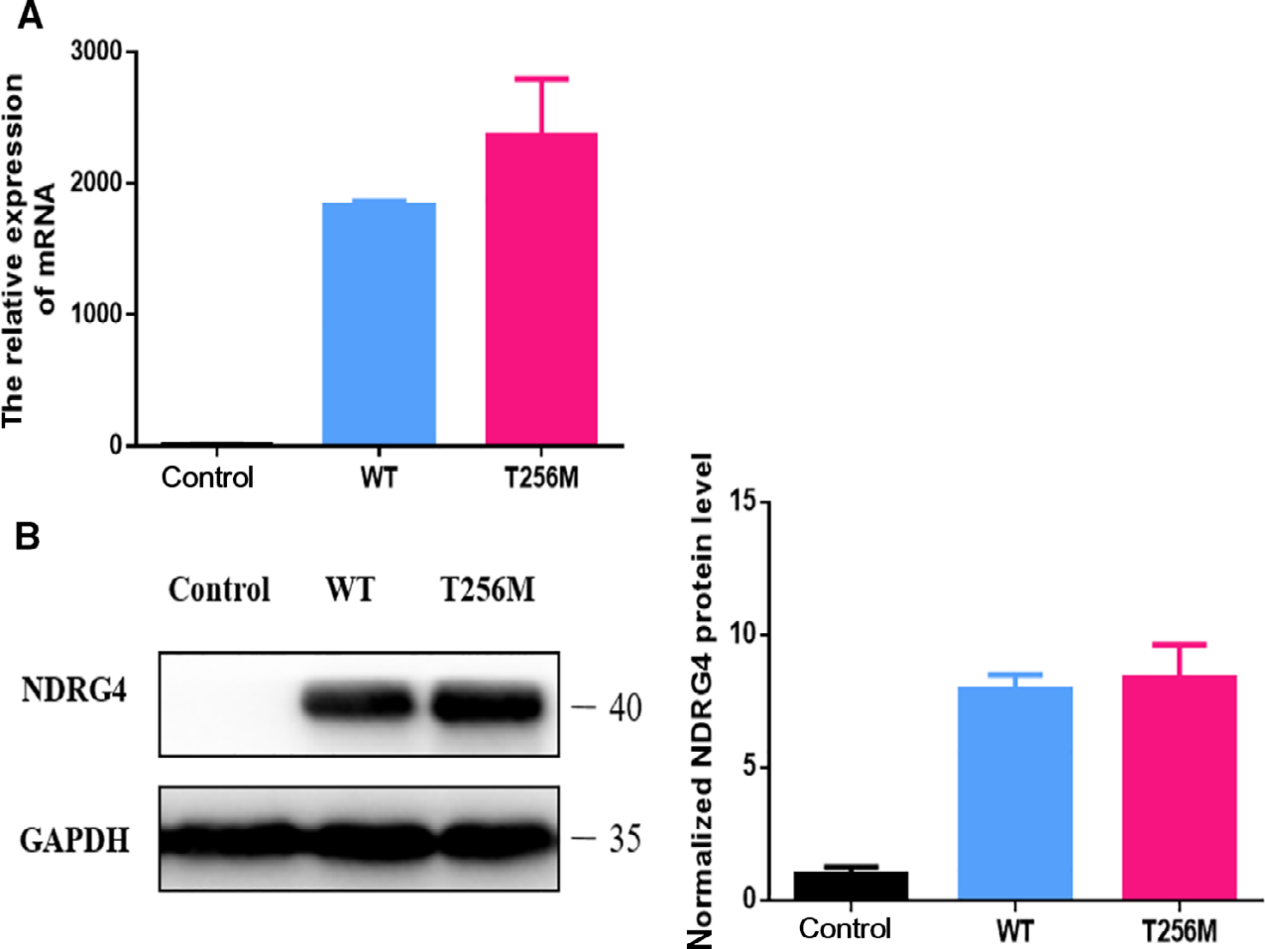
mRNA abundance and protein expression level of NDRG4. hCM was transfected with blank vector (control), as well as WT and variant plasmids of NDRG4, and mRNA and protein expression was analyzed by (A) qRT‐PCR and (B) western blotting (*n* = 4). Data are the mean ± SEM.

To investigate whether the mRNA or protein expression of the NDRG4 variant in hCM was altered, we performed a qRT‐PCR and western blotting. Both experiments found no significant differences in the mRNA and protein expression between WT and p.T256M variant of NDRG4 plasmid (Fig. 3A,B).

### Mutant NDRG4 protein impaired the regulation of cell proliferation in hCM

**Fig. 4:**
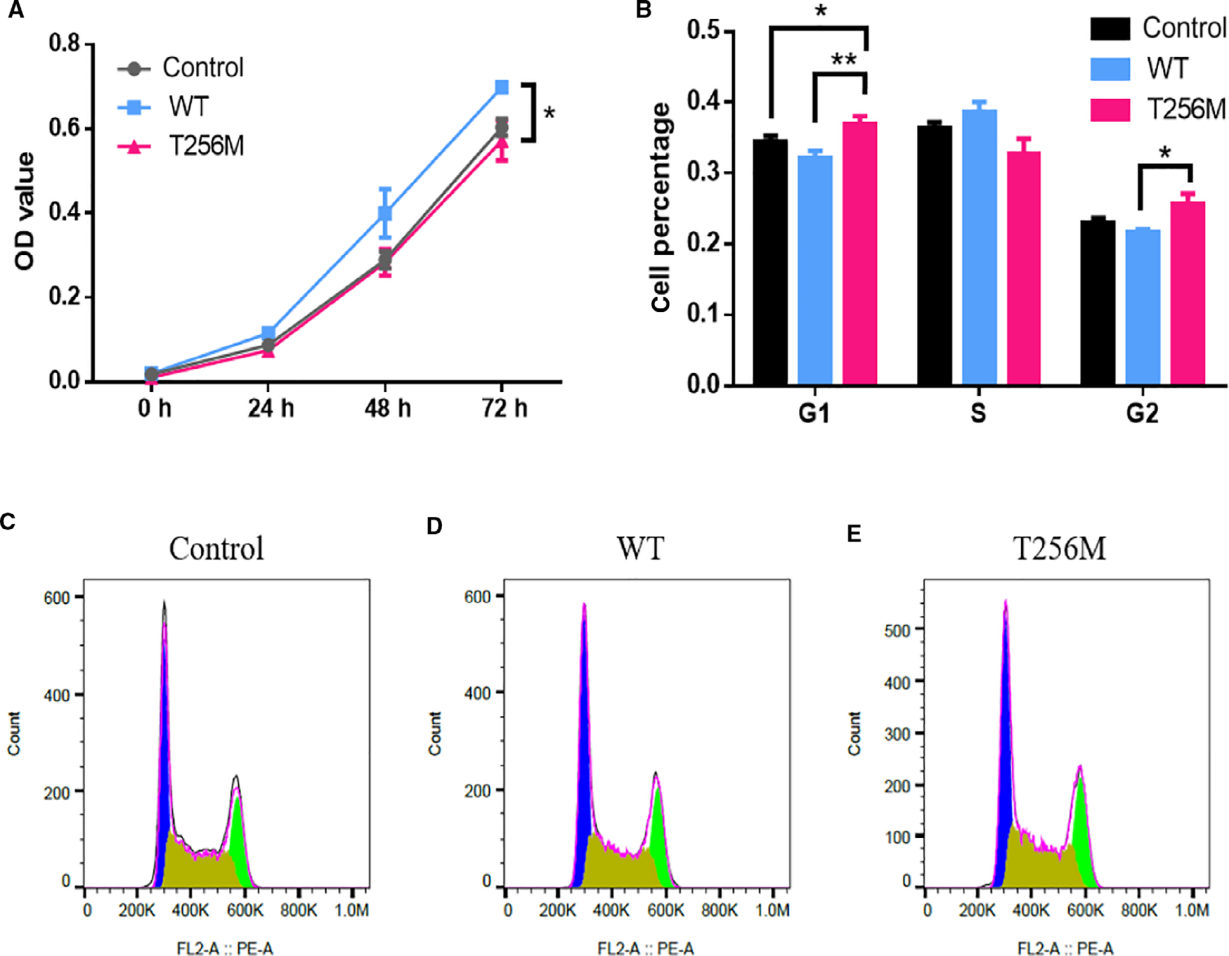
Mutant NDRG4 protein impaired the regulation of cell proliferation in hCM. hCM was transfected with blank vector (control), as well as WT and variant plasmids of NDRG4. (A) Cell proliferation was detected by the CCK‐8 assay at each of the indicated time points (0, 12, 24, 48 and 72 h); two‐way analysis of variance. (B–E) Cell‐cycle distribution was detected using a flow cytometer (*n* = 4, *P < 0.05, **P < 0.01); unpaired two‐tailed Student's *t*‐test. Data are the mean ± SEM.

It was reported previously that NDRG4 knockdown resulted in a marked reduction in myocyte proliferation [19]. To investigate whether this variant will have an impact on the proliferation of hCM or not, we performed the CCK‐8 assay. Our data revealed that, compared to the WT NDRG4 protein, the p.T256M variant showed significant inhibition with respect to the proliferation of hCM (Fig. 4A). We further assessed the effect of mutant NDRG4 protein on cell‐cycle distribution using flow cytometry. Compared to the group of WT NDRG4, hCM was significantly increased at G1 and G2 phase in the p.T256M variant group (Fig. 4B–E).

### The expression and subcellular localization of P27 in hCM

**Fig. 5:**
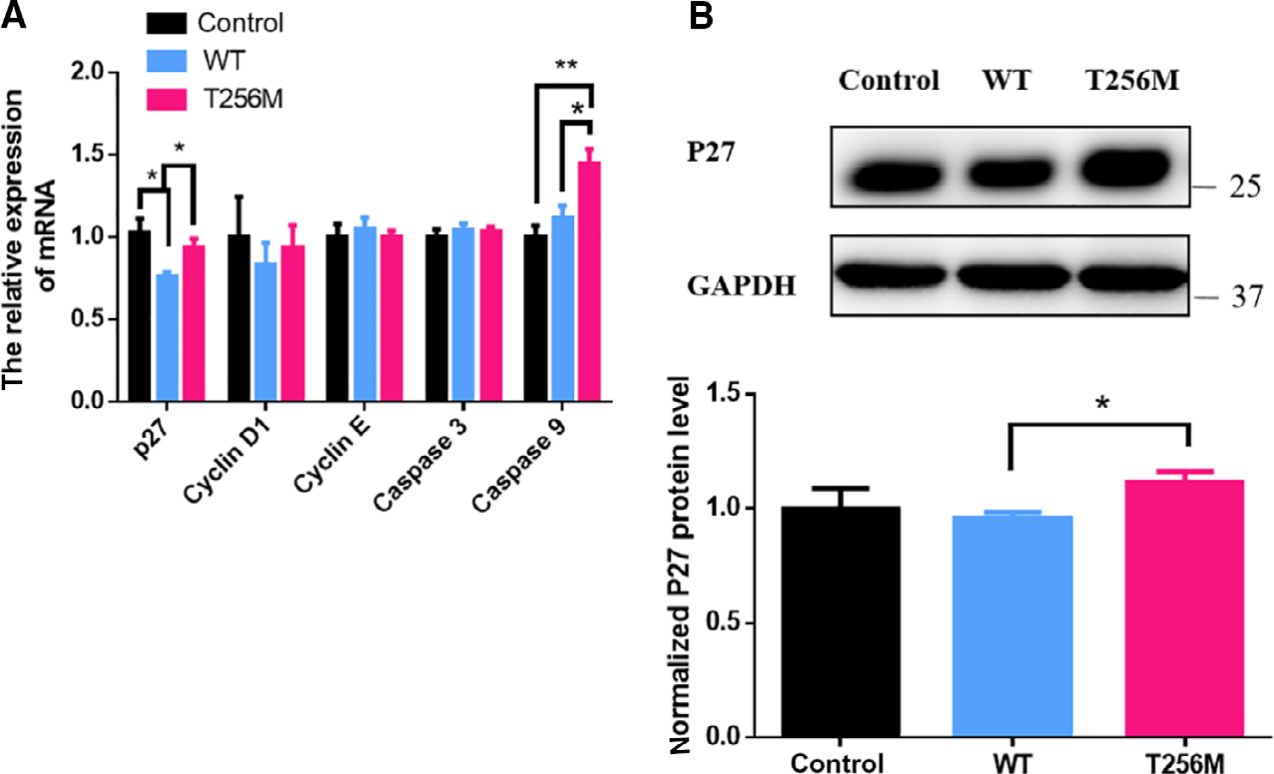
mRNA and protein expression of P27. Blank vector (control), as well as WT and variant plasmids of NDRG4, were transfected into hCM and harvested. (A) qRT‐PCR analysis of cell‐cycle progression markers and apoptotic markers (*n* = 4–5, *P < 0.05, **P < 0.01); unpaired two‐tailed Student's *t*‐test. (B) Western blot analysis of P27 (*n* = 3); unpaired two‐tailed Student's *t*‐test. Data are the mean ± SEM.

**Fig. 6:**
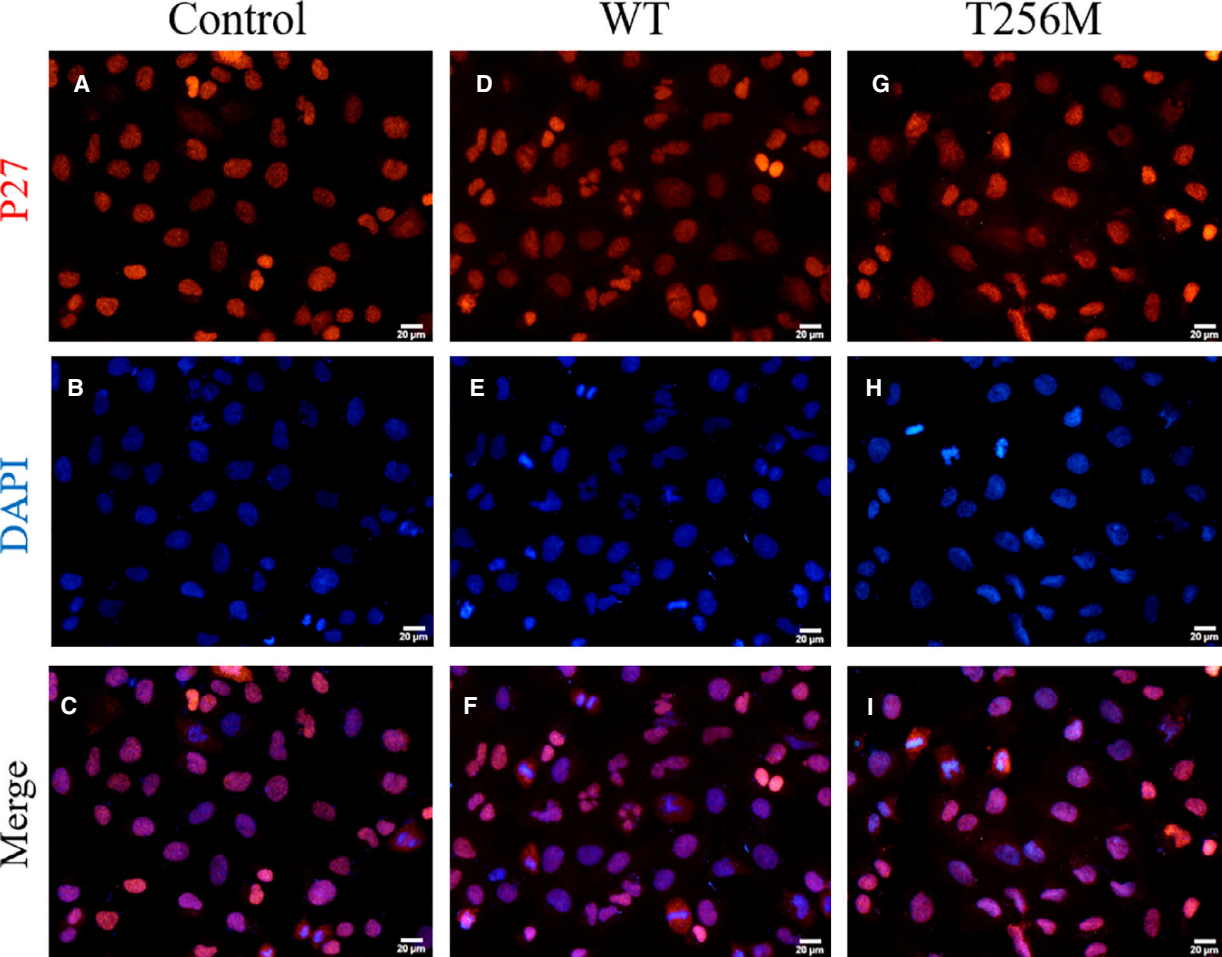
Subcellular localization of P27. Blank vector (control), as well as WT and variant plasmids of NDRG4, were transfected into hCM and harvested. (A–I) Representative images of immunofluorescence staining of P27 proteins in control (A–C), WT (D–F) and variant (G–I) groups (*n* = 3). p27. Scale bar = 20 μm.

We conducted the qRT‐PCR and western blotting to investigate the expression of cell‐cycle regulators and apoptotic biomarkers. We found that the p.T256M variant increased the mRNA expression of P27 and caspase‐9 and the protein expression of P27 (Fig. 5A,B), which indicated that this variant resulted in cell‐cycle arrest and apoptosis. However, there were no significant differences in the mRNA expression of cyclin D1 and E between the WT and mutant NDRG4 groups (Fig. 5A). The immunofluorescence staining assays revealed no significant differences with respect to the subcellular localization of P27 among the three groups: control, WT and mutant NDRG4 groups (Fig. 6A–I).

## Discussion

Pulmonary atresia/VSD comprises a conotruncal defect (CTD), which is characterized as a group of congenital cardiac outflow tract anomalies [27]. PA/VSD shares many similar structural and pathological features with TOF [28] and is considered to be the most extreme form of TOF patients [5, 6]. Previous evidence indicates that cardiomyocyte proliferation shapes cardiac structures including chamber formation and ventricular wall growth and mature [7, 29]. As a result, the abnormal cardiomyocyte proliferation will lead to heart defects. In the present study, a heterozygous and nonsynonymous variant of NDRG4‐p.T256M was identified in 80 unrelated PA/VSD or TOF patients, but not in controls. The variant NM_020465: c.767C>T (p.T256M) is referred to as rs144494221 with an allele frequency of 0.001. A cross‐species alignment of multiple NDRG4 protein sequences showed that the p.T256M variant occurs in the highly evolutionarily conserved residues. Further functional analysis revealed that the p.T256M variant repressed cell proliferation in hCM along with cell‐cycle arrest, which may take part in the pathogenesis of PA/VSD and TOF.

Previous studies have reported that NDRG4 expression was abundant in the human heart [16, 30] and the E14.5 murine heart [20]. However, no study has investigated NDRG4 expression in the human embryo at the period of cardiac development. Our data showed that, at CS13 in human embryos, which is the crucial period of the OFT formation, NDRG4 protein was widely expressed in the atrium, ventricle and OFT. This indicated that NDRG4 might be important for human cardiac development. Consequently, we found out that the p.T256M variant repressed cell proliferation in hCM compared to the WT group. This was consistent with the previous finding that NDRG4 knockdown in zebrafish embryos caused a marked reduction in proliferative myocytes [19]. The above findings suggested that NDRG4 played an important role in the proliferation of cardiomyocytes.

How does the p.T256M variant in NDRG4 affect the hCM proliferation? Zhu *et al*. [31] revealed that NDRG4 promoted myogenesis via Akt/CREB activation. Shinichi *et al*. found that NDRG4 modulates mitogenesis via ERK1/2 signaling [17]. However, both AKT and ERK1/2 signaling appeared to remain unaffected when variant NDRG4 was overexpressed in hCM (Fig. S2). Then, we found out that P27, an important cyclin‐dependent kinase inhibitor, was reduced when WT NDRG4 was overexpressed and returned to normal when the variant NDRG4 was overexpressed. These results indicated that NDRG4 might affect hCM proliferation via the regulation of P27.

Cell‐cycle progression is controlled by the complex interaction between cyclin, cyclin‐dependent kinase and cyclin‐dependent kinase inhibitor. Cyclin‐dependent kinase inhibitor P27 is a factor that inhibits the progression of the cell cycle to block cell proliferation [32]. The function of P27 is regulated by expression and subcellular localization. In cancer cells, the nuclear expression of P27 leads to growth inhibition [33], whereas plasma P27 increases the resistance to apoptosis [34]. However, in the present study, the subcellular localization of P27 did not alter, whereas mRNA and protein expression of P27 changed. This implied that the p.T256M variant regulated the P27 function via its mRNA and protein expression but not subcellular localization. This is consistent with a previous study reporting that NDRG4 knockdown causes G1 arrest and an increase of P27 expression in glioblastoma multiforme cells [35].

In addition to the role of P27 in cell‐cycle regulation, P27 as a regulator of cyclin‐dependent kinases/cyclins also affects apoptosis by regulating the activity of these molecules [32]. Upregulation of P27 is associated with G2/M phase cell‐cycle arrest followed by apoptosis of cells [23]. Furthermore, arrest in late G1 or S phase can accelerate apoptosis [8, 36, 37]. Caspase‐9 is a member of caspase family of cysteine proteases, which is an essential initiator caspase required for apoptosis signaling [38, 39]. In the present study, flow cytometry showed that hCM was significantly increased at the G1 and G2 phase in the p.T256M variant group compared to the WT group. Moreover, we found that the p.T256M variant increased P27 and caspase‐9 expression. The above results indicated that the p.T256M variant might cause G1 and G2 arrest followed by apoptosis via P27 and caspase‐9 expression.

However, the present study has some limitations. First, all of the functional assays were performed *in vitro*, whereas transgenic animal models would be helpful to confirm the potential function of this variant. Moreover, the specific mechanism of how the p.T256M variant regulated the expression of P27 needs to be clarified in the future.

In conclusion, the p.T256M variant in NDRG4 impaired the regulation of NDRG4 in the proliferation of hCM, associated with cell‐cycle arrest and apoptosis. Therefore, this variant is a pathogenic variant that might be involved in the pathogenesis of PA/VSD and TOF.

## Conflict of interests

The authors declare that they have no conflicts of interest.

## Data accessibility

The original immunoblots data are provided in the supporting information. All other data supporting the findings of the present study are available from the corresponding author on reasonable request.

## Author contributions

KS, AFC and QW conceived and designed the study. ZM and JP prepared an analytical plan for clinical data and also analyzed the clinical data. JP drafted the initial manuscript. WS and SZ were involved in data collection. JW and QW collaborated with respect to the revision and interpretation of the data and results. All authors reviewed and revised the manuscript, and approved the final version submitted for publication. All authors agree to be accountable for all aspects of the work.

## Supporting information


**Fig. S1.** Protein expression level of AKT and ERK1/2 signaling.
**Fig. S2.** Raw immunoblots.
**Table S1.** qRT‐PCR primer sequences.
