# Supplementary material for: A loss‐of‐function mutation p.T256M in NDRG4 is implicated in the pathogenesis of pulmonary atresia with ventricular septal defect (PA/VSD) and tetralogy of Fallot (TOF)
Source: FEBS Open Bio. 2021 Jan 9;11(2):375–85. doi: 10.1002/2211-5463.13044 (PMC7876499; doi:10.1002/2211-5463.13044)
Supplement: Supplementary file 1 — Fig. S1. Protein expression level of AKT and ERK1/2 signaling. Fig. S2. Raw immunoblots. Table S1. qRT‐PCR primer sequences. [file FEB4-11-375-s001.pdf]

Table Supplement 1. Real-time quantitative PCR primer sequences

Gene	Forward (5′→ 3′)	Reverse (5′→ 3′)
NDRG4	TTGTGGTGTGTCACGTGGAT	TTGAACCCGAAATGCTGCAC
GAPDH	TTGCCCTCAACGACCACTTT	TGGTCCAGGGGTCTTACTCC
P27	TAATTGGGGCTCCGGCTAACT	TGCAGGTCGCTTCCTTATTCC
Cyclin D1	GCTGCGAAGTGGAAACCATC	CCTCCTTCTGCACACATTTGAA
Cyclin E	GCCAGCCTTGGGACAATAATG	CTTGCACGTTGAGTTTGGGT
Caspase 3	CATGGAAGCGAATCAATGGACT	CTGTACCAGACCGAGATGTCA
Caspase 9	CTCAGACCAGAGATTCGCAAAC	GCATTTCCCCTCAAACTCTCAA

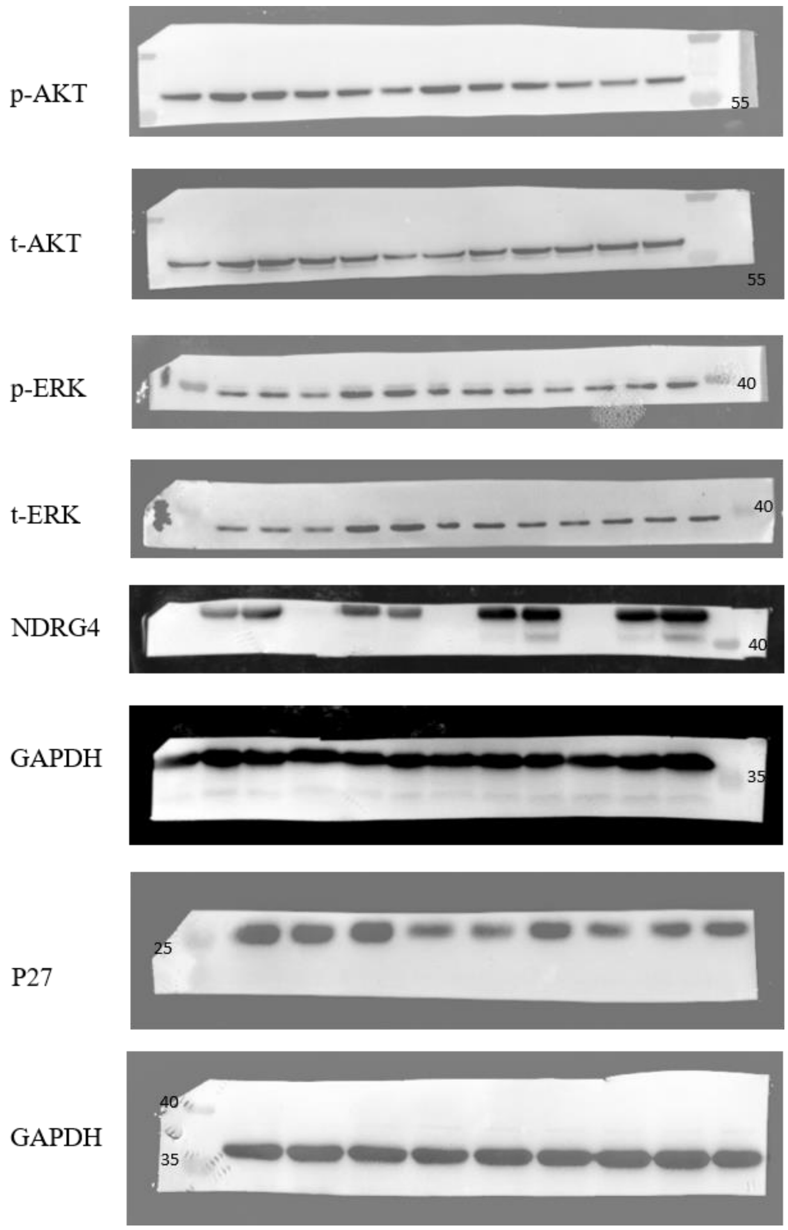

Figure Supplement 1. Raw files of immunoblots.

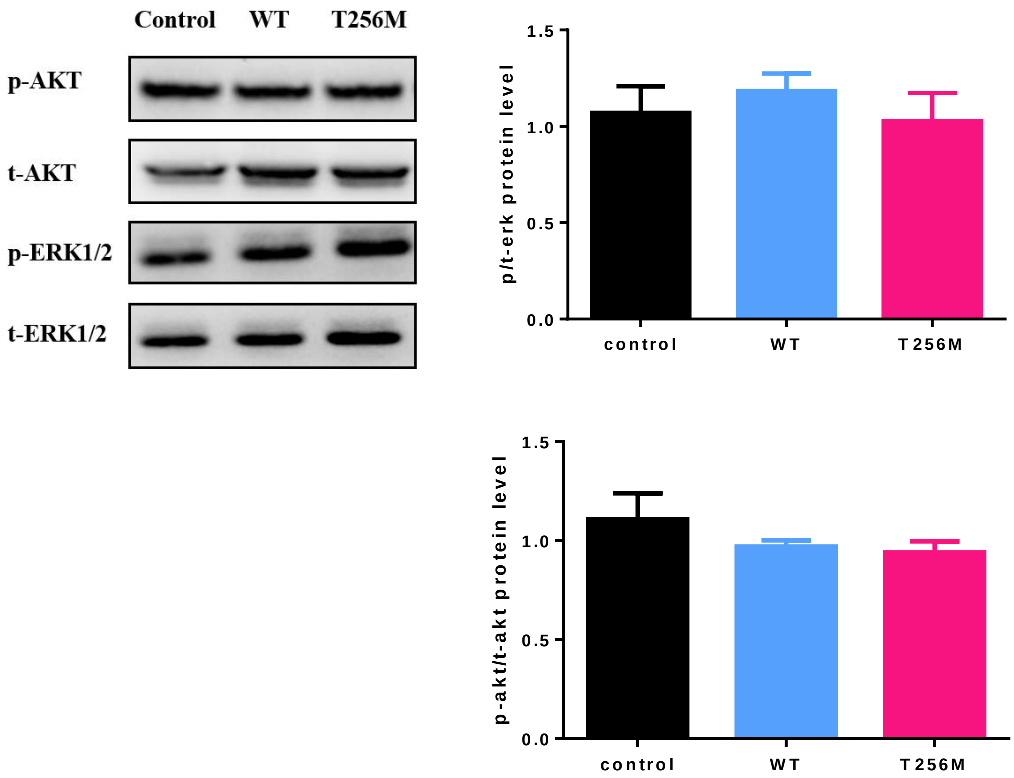

Figure Supplement 2. protein expression level of AKT and ERK1/2 signaling. Western blot analysis of the phospho-AKT (p-AKT), total-AKT (t-AKT), phospho-ERK1/2 (p-ERK1/2) and total ERK1/2 (t-ERK1/2) in the blank vector (control), wild-type, and variant of NDRG4 group (n = 4). Data were represented as mean ± standard error of the mean (SEM).
